# CNN-based two-branch multi-scale feature extraction network for retrosynthesis prediction

**DOI:** 10.1186/s12859-022-04904-7

**Published:** 2022-09-02

**Authors:** Feng Yang, Juan Liu, Qiang Zhang, Zhihui Yang, Xiaolei Zhang

**Affiliations:** Institute of Artificial Intelligence, School of Computer Science, Wuhan University, Wuhan, China

**Keywords:** Retrosynthesis prediction, Convolutional neural network, Machine learning, Multi-scale features

## Abstract

**Background:**

Retrosynthesis prediction is the task of deducing reactants from reaction products, which is of great importance for designing the synthesis routes of the target products. The product molecules are generally represented with some descriptors such as simplified molecular input line entry specification (SMILES) or molecular fingerprints in order to build the prediction models. However, most of the existing models utilize only one molecular descriptor and simply consider the molecular descriptors in a whole rather than further mining multi-scale features, which cannot fully and finely utilizes molecules and molecular descriptors features.

**Results:**

We propose a novel model to address the above concerns. Firstly, we build a new convolutional neural network (CNN) based feature extraction network to extract multi-scale features from the molecular descriptors by utilizing several filters with different sizes. Then, we utilize a two-branch feature extraction layer to fusion the multi-scale features of several molecular descriptors to perform the retrosynthesis prediction without expert knowledge. The comparing result with other models on the benchmark USPTO-50k chemical dataset shows that our model surpasses the state-of-the-art model by 7.4%, 10.8%, 11.7% and 12.2% in terms of the top-1, top-3, top-5 and top-10 accuracies. Since there is no related work in the field of bioretrosynthesis prediction due to the fact that compounds in metabolic reactions are much more difficult to be featured than those in chemical reactions, we further test the feasibility of our model in task of bioretrosynthesis prediction by using the well-known MetaNetX metabolic dataset, and achieve top-1, top-3, top-5 and top-10 accuracies of 45.2%, 67.0%, 73.6% and 82.2%, respectively.

**Conclusion:**

The comparison result on USPTO-50k indicates that our proposed model surpasses the existing state-of-the-art model. The evaluation result on MetaNetX dataset indicates that the models used for retrosynthesis prediction can also be used for bioretrosynthesis prediction.

## Introduction

In the field of organic chemistry and drug development, it is often necessary to find a series of reactants to synthesize a target compound. It originated from the synthesis of tropinone [1], which was later formalized as retrosynthesis prediction [2] and has become one of the fundamental problems in organic chemistry. The huge search space of the retrosynthesis prediction problem makes it difficult for traditional approaches (physical organic, quantum chemistry, etc.) to solve such problems. For decades, researchers have started to use computer techniques to assist the retrosynthesis prediction [3]. Owing to the fact that machine learning has made significant progress in some filed, such as computer vision and text classification, researchers have begun to use machine learning in retrosynthesis prediction.

For this purpose, several prediction models have been proposed, such as RetroSim [4], NeuralSym [5], Seq2Seq [6], and [7], etc., utilize machine learning to assist in prediction tasks. Existing machine learning-based models for retrosynthesis prediction can be roughly classified into two categories: template-based models as well as template-free models. The models in the first category utilize reaction templates to aid the prediction process, while models in the second category consider retrosynthesis prediction as a sequence-to-sequence translation problem.

Most existing models mainly use molecular descriptors as a whole for retrosynthesis prediction. For example, molecular fingerprints (Extended Connectivity Fingerprints, ECFP) convert a compound molecule into a string of binary vectors that are characterized by the presence or absence of a certain type of molecular fragment and are usually used to compare the similarity of molecular fingerprints to select a suitable template, or by directly inputting a deep neural network (DNN) as a feature to predict the template. For other descriptors, such as SMILES, which utilizes simple atomic symbols, bond symbols, and linguistic rules to describe the three-dimensional structure of a molecule, it is often used in seq2seq models to obtain a direct translation of products to reactants. However, these existing models either do not capture the full exploitation of the multiple descriptors of the molecule, resulting in low prediction accuracy, or improve the prediction accuracy by building complex models, resulting in high time consumption. As mentioned above, SMILES focuses on global information, while molecular fingerprinting focuses on molecular structure information, both of these two descriptors are the structured representation, and descriptors already contain high-dimensional features of molecules, such as functional group features. The absence of either of these descriptors will result in the absence of molecular features. In addition, the features for these descriptors are also not fully utilized, such as multi-scale features.

Therefore, in this paper, we utilize several molecular descriptors, extract the multi-scale features of these descriptors using CNN [8] and leverage these features for retrosynthesis prediction. Specifically, the main contributions of this paper are as follows:

- We propose a new CNN-based multi-scale feature extraction network. The proposed network concatenates several filters of different sizes. Through this design consideration, the proposed network is able to adaptively extract the multi-scale features of molecular descriptors.
- We propose an end-to-end model, named CNN-TMN, based on the above network for retrosynthesis prediction. CNN-TMN consists of a two-branch feature extraction layer to extract scalable features of different molecular descriptors and uses these features for retrosynthesis prediction.
- We validate the superiority of CNN-TMN on the standard USPTO-50k derived from a patent database [9] and get the accuracies of Top-1 and Top-10 of 61.1% and 87.7%, respectively. We also validate CNN-TMN on the MetaNetX datasets as the first attempt in the field of bioretrosynthesis prediction and provide a baseline for the metabolic reaction dataset.

## Related work

In this paper, a reaction refers in particular to the synthesis reaction in which two or more molecules collide and react with each other to produce a new molecule. For example, the reaction $A + B \xrightarrow []{}C$ indicates that molecules A and B synthesize a new molecule C. A and B are called as reactants, and C is called as the product. Retrosynthesis is the process of decomposing the target product into building blocks (reactants). The purpose of retrosynthesis prediction is to predict the reactions that can produce the target product. The existing methods are mainly divided into two categories: template-free and template-based methods. We first introduce the template-free methods in “Template-free methods” section; and then introduce the related template-based methods in “Template-based methods” section.

### Template-free methods

The template-free methods usually build models with known reactions to predict the reactants of the target product. The built models can then be used to directly translate the target product (represented with the molecular descriptors such as sequence and graph) into the reactants. The first template-free model is Seq2Seq, that was built based on Recursive Neural Network (RNN) [6]. After that, Karpov et al. built a Transformer-based method [10] and achieved better prediction performance than Seq2Seq. Tetko et al. proposed to use data augmentation strategy to train the model based on investigating the effect of Transformer with different training scenarios and further improve the performance [11]. As the chemical molecules are often represented as the molecular graph, some researchers begin to leverage graph information to enhance the prediction accuracy. For example, Seo et al. [12] leverages both sequence and graph to improve the performance of Transformer. Shi et al.[13] utilizes the molecular graph information to formulate the retrosynthesis prediction as the transformation of graph-to-graph problem. Different from the previous template-free models, Hasic et al. utilize molecular fingerprint to generalize knowledge directly from the structure of a target molecule without additional information. Due to the lack of the guidance by templates, the prediction model can not reveal the reaction relationship between reactants and products, which reduces the reliability of prediction results.

### Template-based methods

In template-based methods, the reactions are organized into a set of templates according to their atomic mapping information to describe the transformation relations between the reactants and the corresponding products. The templates can be either hand-crafted by human experts, or automatically extracted from a large number of known reactions by developing a toolkit such as RDKit [14]. Therefore the main objective of retrosynthesis prediction in template-based methods is to find suitable templates for the target product, for it is very easy to obtain the corresponding reactions from the found templates. Based on the assumption that similar templates produce similar products, Coley et al. proposed a similarity-based method, named RetroSim, which used the molecular similarity as an effective metric to select templates for target products [4]. Obviously, such similarity-based method is very simple and easy to implement. However, its performance is sensitive to the adopted similarity measure. Segler et al. considered the retrosynthesis prediction problem as a multi-classification problem where each class corresponds to one template, and built an MLP (multi-layer perception) based model, named NeuralSym, to predict the templates of the target product [5]. This method can automatically learn the nonlinear relationships between the molecular ECFP fingerprints and the templates via a series of hidden layers without any background knowledge, thus easy to implement. However, it suffers from two drawbacks. Firstly, there is a lack of convolutional layers in NeuralSym, resulting in that it can not learn template-related features that are benefit for the prediction. Secondly, the molecule representation with the ECFP fingerprint can only describe the presence or absence of a structural unit in the molecule, incapable of capturing the unit-unit interactions and detailed structure information. Recently, several researchers proposed to build prediction models based on graph learning. For example, Dai et al. proposed a conditional graphical model based on graph neural networks (GNNs), named GLN, to predict the conditional probabilities of the templates for the target product, implicitly considering the chemical feasibility and strategy of the corresponding reactions [15]; Somnath et al. taking the retrosynthesis prediction problem as the one that identifies precursor molecules that can be used to synthesize a target product, and proposed graph-based approach, GraphRetro, based on the idea that the graph topology of precursor molecules is largely unaltered during a chemical reaction [7]. Compared to other methods, the graph-based methods embed the domain knowledge of the reactions into the graph models thus can achieve superior performance. However, they mainly rely on the operation of subgraph isomorphism thus have poor scalability. Moreover, heavy dependency on the domain knowledge makes it difficult to build a robust graph model for beginners.

## Proposed method

Our method belongs to the template-based methods. Similar to the idea of [5], we also consider each template as a class, and the task of retrosynthesis prediction as a multi-classification problem accordingly. Different from [5], we design a sophisticated features extraction network to learn multi-scale features beneficial to the classification. Before introducing the details of our method, we present the definition of the problem.

### Problem definition

For a reaction with *M* reactants $R_i: S_{i,1}+\cdots +S_{i,j}+\cdots S_{i,M} \longrightarrow P_i$, we use the following quadruple to represent $R_i$:

1 $$\begin{aligned} R_i=(\{S_{i,j}\}_{j=1}^M,P_i,T_i,C_i) \end{aligned}$$

where $\{S_{i,j}\}$ is the set of reactants of $R_i$, *M* is the total number of reactants, $P_i$ is the product of $R_i$, $T_i$ is the template extracted from $R_i$, and $C_i$ is the class label of $T_i$. In our work, $S_{i,j} (j=1,\dots ,M)$, $P_i$ and $T_i$ are all represented as SMILE sequences. For convenience, we set $C_i$ as a positive integer.

Let the $(P_i, C_i)$ pair denotes a training sample, the objective of the problem is to train a multi-classification model $\theta$ with the distribution $p(C|P, \theta )$ using a set of training samples, where *p* represents the probability that the input product *P* belongs to the template with class label *C*.

### Construction of CNN-TMN model

#### CNN-based feature extraction network

**Fig. 1:**
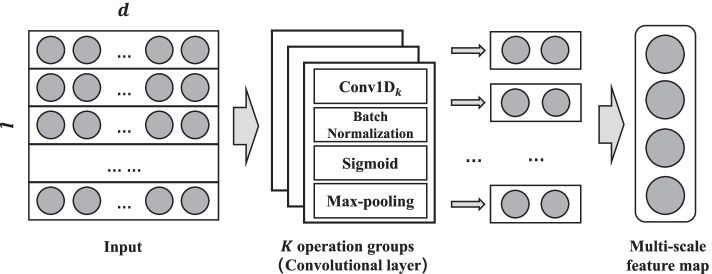
The architecture of CNN-based feature extraction network. The input of the network is the sequence matrix, and the output is the multi-scale feature map. *l* is the length of the input matrix. *d* is the dimension of the input matrix. The **operation groups** contains convolution, batch normalization, activation and max-pooling operations

In this section, we will introduce the proposed feature extraction network. The main idea of our network is to utilize multiple filters of different sizes to extract the scalable features and integrate them to obtain the final multi-scale features. In other word, our network can also be regarded as the concatenation of *K* simple **operation groups**, which contains convolution, max-pooling, batch normalization and activation operations. The architecture of the proposed feature extraction network is shown in Fig. 1. The input of our network is a one-hot matrix, and the output is the multi-scale feature map.

The filters used in our network are all one-dimensional filters [8], termed Conv1D. Let $X\in R^{l\times d}$ be the input of the network, which is a one-hot matrix of the input sequence, where *l* is the length of the sequence, *d* is the dimension of the matrix and equal to the input channel of Conv1D. The *X* will be fed into *K* different operation groups to get the final multi-scale feature map. Within each operation group, the operations are conducted sequentially. First, the pointwise product between the input *X* and filter *F* is conducted to get the extracted feature map after the Conv1D. Then, the extracted feature map is standardized by batch normalization [16] in order to reduce internal covariate shift, improve the generalization by regulating the distribution of the data, and improve the training speed. For the normalized feature map, we will apply the activation function. Here, we use the sigmoid function [17] to map the features in [0, 1]. Later, the max-pooling [18] operation is adopted to downsample the feature map. Finally, the feature maps outputted by different operation groups are concatenated together to get the final multi-scale feature map.

The total number of operation groups, which is the same as the number of different sizes of filters, is determined by a parameter *step*. Suppose the minimum size of filter is *min*, the maximum is *max*, and the step is *step*, the size of the *i*-th filter in *i*-th operation group is calculated as: $F_i = min +(i-1)\times step$, where $F_i < max$.

#### CNN-TMN model

**Fig. 2:**
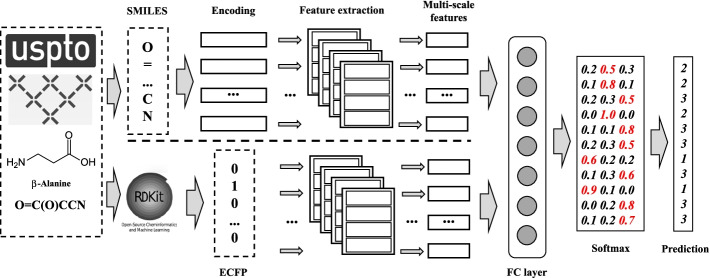
The overview of CNN-TMN. The input of CNN-TMN is chemical molecules, while the output is the predicted label *L*. Firstly, the SMILES and ECFP are canonicalized and extracted using RDKit. Then, these two descriptors are then fed into two different branches of the feature extraction network to extract their multi-scale features respectively. Finally, the concatenated features are fed into the full connection and softmax layer to get the final prediction result

In this section, we will introduce CNN-TMN in detail. The overview of CNN-TMN is shown in Fig. 2. The idea of CNN-TMN is to use a two-branch feature extraction layer based on the proposed network to extract the multi-scale features of molecular descriptors and integrate them to obtain the final prediction result. In this paper, we choose molecular descriptors as molecular fingerprints (specifically ECFP) and SMILES. The specific details of the two different branches used to extract the multi-scale features of the above ECFP and SMILES are described as follows.

For the feature extraction of the ECFP, the input of the network is the ECFP, and the output is its multi-scale feature map. In this paper, ECFP is formed by binary vector: $X=\{x_1,x_2,{\ldots },x_i,\ldots ,x_L\}$, where $x_i\in \{0,1\}$, and *L* is the maximum length of ECFP. Since ECFP is a one-dimensional binary vector, it can be directly used as an input to the feature extraction network. The input channels of all *K* filters are set to 1. The sizes of filters are set from 32 to 2048 with a *step* of 32, that is, the sizes of the filters are $\{32,64,96,\ldots ,2048\}$, a total of 64 filters. After the feature extraction, we will get the multi-scale features of the ECFP.

Before extracting the SMILES multi-scale features, one-hot encoding is used to encode the SMILES sequence. First, the word set *tokens* is introduced. The *tokens* is composed of different characters in the dataset, and for USPTO-50k and MetaNetX, the lengths are 40 and 48, respectively. For a sequence of length *L*, the shape of the one-hot matrix is $L\times length(tokens)$. The length of the SMILES sequence is determined by the distribution of the dataset, which will be introduced in “Dataset splitting strategy” section. After encoding, similar to the feature extraction of ECFP, the one-hot matrix is fed into the feature extraction network to get the multi-scale features of molecular SMILES. Compared with the ECFP, in SMILES, since the dimension of the one-hot matrix is 40, so the input channels for all *K* filters are set to 40. The sizes of all filters are set from 5 to *L* with the *step* of 5, that is, the sizes of the filters in extracting the SMILES features are $\{5,10,15, \ldots ,L\}$, where the maximum size of filters is smaller than *L*.

### Loss function

For the loss function, we use cross entropy loss function:

2 $$\begin{aligned} \begin{aligned} loss=-\frac{1}{N}\sum _{i}\sum _{c=1}^{M}y_{ic}log(p_{ic}) \end{aligned} \end{aligned}$$

where *M* and *N* are the total number of labels and samples, respectively, $y_{ic}$ represents a symbolic function, if the real label of sample *i* is the same as *c*, then $y_{ic}=1$, $p_{ic}$ represents the probability of the predicted label *c*.

## Experiments

We conduct two kinds of experiments to test CNN-TMN. Firstly, we comprehensively evaluate the retrosynthesis prediction performance of CNN-TNM by designing a series of comparison experiments using the benchmark chemical reaction dataset USPTO-50k [9]. Since there is no public report on bioretrosynthesis prediction. Secondly, we attempt to use CNN-TMN for bioretrosynthesis prediction by using the widely used metabolic reaction dataset MetaNetX [19] to investigate its feasibility.

### Datasets and data preprocessing

#### The datasets

The USPTO-50k dataset has been used as a benchmark dataset in previous retrosynthesis prediction works [6, 10]. It currently contains 50,016 items, each corresponds to an atom-mapped reaction denoted by a SMILES sequence. The MetaNetX metabolic reaction dataset contains 30,986 unique metabolic reactions. Same as USPTO-50k, all reactions are denoted as SMILES sequences. The outline of two datasets is listed in Table 1.

**Table 1:** Statistical information of two reaction datasets

Statistical information	Dataset	
	USPTO	MetaNetX
Dataset size	50,016	30,986
Template number	11,856	15,939
Product number	49,676	8,795
Reactant number	64,123	8,616
Maximum product length	708	243
Minimum product length	7	1
Average product length	786.6	37.6

#### Data preprocessing

Since the original datasets are composed of reaction SMILES, we need to process each reaction SMILES sequence to extract every reactant and product and to generate the corresponding template and its class label. Firstly, we sliced each reaction SMILES into reactants and products according to the symbol ”>”. For multi-product reactions, we split them into multiple single-product reactions. Then, we used RDKit[14] to extract the reaction templates of all reactions to form the template set. Considering that multiple reactions may have the same template, we then removed redundant templates from the template set. For each unique template in the set, we assigned a positive integer to it as the class label. Finally, we associate class labels with the corresponding products to construct the dataset $D={(P_i,C_i)}$ ($P_i$ denotes the product, and $C_i$ denotes its class label).

**Fig. 3:**
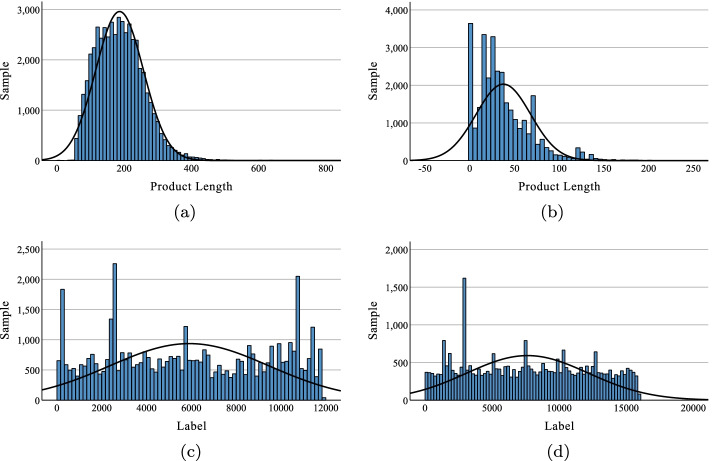
The distribution of SMILES lengths of products in different datasets (**a**). The product lengths distribution in USPTO-50k dataset (**b**). The product lengths distribution in MetaNetX dataset (**c**). The product labels distribution in USPTO-50k dataset (**d**). The product labels distribution in MetaNetX dataset

We statistically analyze the distributions of product length and the number of products in different classes, shown in Fig. 3. From this figure we can see, the majority of the product sequences in USPTO-50k are less than 300, while the majority of the product sequences in MetaNetX are less than 100. As described in “CNN-TMN model section, we need to set the lengths of the products to the same length *L* when performing one-hot encoding. If the length of the product is less than *L*, we will insert zeros and inevitably introduce noise. The larger *L* is, the more product sequences contain noise. Therefore, according to the analysis results, we set *L* to 300 and 100 for USPTO-50k and MetaNetX, respectively. In addition, we find that the total number of different classes are extremely large (11,856 in USPTO-50k, 15,939 in MetaNetX), whereas the number of products per class is rather small (about 5 in USPTO-50k and 2 in MetaNetX on average), which leads to the difficulty of the retrosynthesis prediction problem.

### Experimental setup

#### Baselines

To evaluate CNN-TMN, we refer to eleven comparison baselines, including five template-based models and six template-free models. In specific:

- *Template-Based*: **RetroSim** [4]; **NeuralSym** [5]; **GLN** [15]; **EBMs** [20]; **GraphRetro** [7].
- *Template-Free*: **Seq2Seq** [6]; **Transformer** [10]; **G2Gs** [13]; **Tetko’s** [11]; **GTA** [12]; **Hasic’s** [21].

#### Evaluation metric

We use the widely used Top-*k* ($k=1, 3, 5, 10$) exact match accuracy as our evaluation metric following the previous works [4–6]. This metric compares whether predicted SMILES sequence is the same as the ground truth sequence.

#### Implementation details

CNN-TMN is implemented in PyTorch [22]. The ECFP of molecules and templates of reactions are extracted by the open-source chem-informatics software RDKit [14]. The sizes of filters in ECFP and SMILES features are set from 32 to 2048 with a step of 32 and 5 to 200 with a step of 5, respectively. All the lengths of molecular sequences are set to 300 for one-hot encoding. CNN-TMN is trained for 20 epochs with a batch size of 128 and a learning rate of 0.001 with Adam [23] optimizer on a single NVIDIA RTX 2080Ti GPU. We take approximately one hour to train CNN-TMN.

#### Dataset splitting strategy

To train CNN-TMN, we utilize a splitting strategy commonly used in this field [6], the training/validation/test set is randomly divided into 80%/10%/10%, termed ”Plain”. In addition, since we consider the retrosynthesis prediction problem as a multi-classification problem, the splitting strategy can have an impact on the final prediction performance of the model. The splitting strategy used in [6] can lead to the label imbalance. Therefore, we design a new splitting strategy termed “Aug” as the data augmentation. The new splitting strategy ensures that each label in the training set will appear at least once, and the splitting ratio is the same as in the previous work.

### Evaluation results on UPSPTO-50k dataset

#### Performance comparison of different models

This section compares the model proposed in this paper with the eleven retrosynthesis models on the USPTO-50k benchmark dataset, and the experimental result is shown in Table 2. All the comparison models use the same splitting strategy.^1^

**Table 2:** Comparisons of average accuracies (%) against state-of-the-art models for retrosynthesis on the USPTO-50k dataset

Model	Top-*k* accuracy %			
	1	3	5	10
*Template-free*				
Seq2Seq	37.4	52.4	57.0	61.7
Transformer	42.7	63.9	69.8	$\setminus$
Hasic’s	47.2	55.7	61.5	65.1
GTA	47.3	67.8	73.8	80.1
G2Gs	48.9	67.6	72.5	75.5
Tetko’s	53.5	69.4	81.0	85.7
*Template-based*				
RetroSim	37.3	54.7	63.3	74.1
NeuralSym	38.5	55.7	61.3	66.6
GLN	52.5	69.0	75.6	83.7
GraphRetro	53.7	68.3	72.2	75.5
EBMs	55.2	74.6	80.5	86.9
CNN-TMN (Plain)	49.1	64.4	67.6	72.6
CNN-TMN (Aug)	**61.1**	**79.1**	**83.9**	**87.7**

The best result are indicated in bold

Where the suffix (Plain) and (Aug) indicate two different splitting strategies proposed in “Dataset splitting strategy” section

After using a reasonable splitting strategy, CNN-TMN has 7.6% higher prediction accuracy compared to the template-based state-of-the-art model GraphRetro, and 7.4% higher compared to the template-free model Tetko’s. Moreover, the performance improvement in the prediction accuracy is also seen with the increase of *k*, especially when $k=10$. Among the compared models, NeuralSym is similar to CNN-TMN, which also considers the retrosynthesis prediction problem as a multi-classification problem. CNN-TMN is 10.6% higher than it in terms of accuracy and 22.6% higher after correct splitting. The experiment result proves the effectiveness of CNN-TMN for retrosynthesis prediction.

As mentioned in “Dataset splitting strategy” section, the splitting strategy has a large impact on the performance of CNN-TMN. When the splitting strategy is not reasonable, there is a large degradation in the prediction performance of CNN-TMN and the accuracy of CNN-TMN decreases by 12% in Top-1 accuracy. In [6], the dataset is spitted randomly, while in this paper, we split the dataset with a new strategies, which is introduced in “Dataset splitting strategy” section. In order to explore the impact of different splitting strategies on the prediction accuracy, we chose the NeuralSym, which is similar to ours for the performance comparison under different splitting strategies, and the result is shown in Table 3.

**Table 3:** Comparisons of average accuracies (%) against two different strategies for retrosynthesis on the USPTO-50k dataset

Model	Top-*k* accuracy %			
	1	3	5	10
NeuralSym (Plain)	38.5	55.7	61.3	66.6
NeuralSym (Aug)	**45.4**	**67.7**	**73.5**	**81.1**
CNN-TMN (Plain)	49.1	64.4	67.6	72.6
CNN-TMN (Aug)	**61.1**	**79.1**	**83.9**	**87.7**

The best result are indicated in bold

The (Plain) and (Aug) indicate two different splitting strategies, which have been introduced in “Dataset splitting strategy” section

The results in Table 3 show that the splitting strategy has a very significant impact on the performance of both CNN-TMN and NeuralSym. The main reason is that random splitting will lead to class imbalance, and if a particular label is not included in the training set, the prediction accuracy for that class will be greatly reduced. After a reasonable splitting, the accuracies of both have been improved significantly. For NeuralSym, the prediction accuracy is improved by 6.9% after using a reasonable splitting strategy, while for CNN-TMN, the prediction accuracy is improved by 12%. The above experiments demonstrate that our splitting strategy can significantly improve the prediction accuracy of this type of model.

#### Ablation study

The ablation study aims to verify the effectiveness of using multiple descriptors and the proposed feature extraction network. First, we explore the effectiveness of using different molecular descriptors, and the result is summarized in Table 4. The check $\checkmark$ in the table indicates that the CNN-TMN uses a certain molecular descriptor. For example, the first row indicates that the CNN-TMN only utilizes ECFP for prediction.

**Table 4:** Ablation study on the effectiveness of using different molecular descriptors. ECFP and SMILES indicate that CNN-TMN only uses ECFP or SMILES descriptor for retrosynthesis prediction

ECFP	SMILES	Top-1	Top-3	Top-5	Top-10
$\checkmark$	–	47.2	67.6	73.5	80.0
–	$\checkmark$	53.8	73.1	78.7	84.8
$\checkmark$	$\checkmark$	**61.1**	**79.1**	**83.9**	**87.7**

The best result are indicated in bold

By only using the ECFP descriptor, as shown in the first row, it achieves 47.2% Top-1 accuracy and 80.0% Top-10 accuracy. By only using the SMILES descriptors of molecules, we achieve a 53.8% Top-1 accuracy and 84.7 Top-10 accuracies. By further fusing the two descriptors, we obtain a final Top-1 accuracy of 61.1%, as shown in the last row. The above experimental result demonstrates that using multiple molecular descriptors can significantly improve the accuracy of prediction.

Then, we verify the effectiveness of our proposed feature extraction network in extracting the multi-scale features of molecular descriptors. The result is shown in Table 5. As we can see in Table 5 indicates that the proposed network in this paper can effectively extract the multi-scale features of the molecular descriptors and improve the prediction accuracy by 26.65% on average compared to the baseline. Also, for the two different molecular descriptors, the impact on the accuracy is different, and overall, the accuracy using SMILES is 1.6% higher than that of ECFP. The main reason is that the ECFP descriptor is a one-dimensional vector in presentation, and compared with SMILES, ECFP is sparser in features, which leads to the different performance of the multi-scale feature extraction network in extracting two molecular descriptors.

**Table 5:** Ablation study on the effectiveness of using feature extraction network for different molecular descriptors. FE and no FE indicate whether we use the proposed feature extraction network

Model				Top-k accuracy %			
ECFP		SMILES		1	3	5	10
FE	No FE	FE	No FE				
–	$\checkmark$	–	$\checkmark$	25.1	40.8	46.8	57.2
–	$\checkmark$	$\checkmark$	–	51.0	70.5	75.3	82.9
$\checkmark$	–	–	$\checkmark$	48.9	68.9	74.5	81.1
$\checkmark$	–	$\checkmark$	–	**61.1**	**79.1**	**83.9**	**87.7**

The best result are indicated in bold

To further validate the effectiveness of the proposed feature extraction network, we replace the network with a commonly used CNN architecture TextCNN [8], which is commonly used in the field of text classification, and the rest of the network remains unchanged. The experiment result is shown in Table 6. Compared with TextCNN, CNN-TMNl has a significant improvement, mainly because we use different sizes of filters to extract features of different scales, while in TextCNN, the maximum filter is only 5. By using large convolutional kernels, we are able to capture different length dependencies of the sequence, which can better improve the prediction accuracy of the model.

**Table 6:** Comparisons of average accuracies (%) against two feature extraction network for retrosynthesis on the USPTO-50k dataset

Model	Top-k accuracy %			
	1	3	5	10
TextCNN	21.6	39.9	43.9	53.9
CNN-TMN	**61.1**	**79.1**	**83.9**	**87.7**

The best result are indicated in bold

#### Influence of the *step* on the performance

**Fig. 4:**
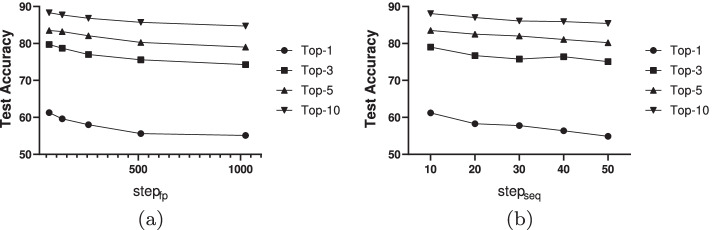
The comparison accuracies (%) obtained by different *step*. **a** The $step_{fp}$ for the extraction of ECFP features, **b** the $step_{seq}$ for the extraction of SMILES sequence features

In CNN-TMN, we have one parameter *step* to investigate, which is used to determine the size of filters in the feature extractions of SMILES and ECFP. This section will discuss the impact of this parameter in detail. For the filters used to extract ECFP $step_{fp}$ and SMILES $step_{seq}$, they are set as $\{64,128,256,512,1024\}$ and $\{10,20,30,40,50\}$, respectively. The result is shown in Fig. 4.

As we can see in Fig. 4, both in two figures, higher accuracy can be obtained by using a smaller *step*, and vice versa. A larger *step* will lead to coarse-grained features, while a smaller *step* can make the extracted features more fine-grained. Even if the fingerprint is sparse, the features can also be extracted by using filters of different sizes. However, the disadvantage of a smaller *step* is that the increase of the number of filters will make the architecture of the network more complex, thus increasing the computational cost. This result supports the hypothesis that by using filters of different sizes, we can make good use of CNN to extract the multi-scale features of sequences.

### Model complexity analysis

As mentioned above, there is a parameter *step* that controls the number of operation groups in the feature extraction network. To study the effect of the different *step* on the model size, we add a statistical experiment on the number of model parameters under different *step*. Since the steps used for extracting different molecular descriptors are different, the number of model parameters under different *step* are listed separately, and the statistical result is shown in Table 7.

**Table 7:** Statistical result of the number of parameters in CNN-TMN at different *step*

Parameters	$step_{seq}$					
	5	10	20	40	80	160
	32,479,537	15,988,017	7,742,257	3,619,377	1,557,937	527,217
	$$step_{fp}$$					
	32	64	128	256	512	1024
	12,382,657	6,038,657	2,866,657	1,280,657	678,257	377,057

The statistical results in the table show that the number of parameters in CNN-TMN is negatively correlated with the parameter *step*, and both decrease with the increase of *step*. Meanwhile, together with the result in Fig. 4, it can be found that the size of *step* is also negatively correlated with the accuracy of CNN-TMN. The main reason is that when we use a smaller *step*, CNN-TMN can utilize more parameters to characterize the molecular descriptors, thus improving the prediction accuracy.

### Feasibility investigation for bioretrosysthesis prediction

Since retrosynthesis prediction is focused on chemical reaction prediction because of the simplicity of chemical reactions, but in biological systems, metabolic reactions are more complex than chemical reactions, as far as we know, there is no published work attempted on the metabolic reaction dataset such as MetaNetX. In order to verify the performance of CNN-TMN in bioretrosynthesis prediction, we apply CNN-TMN to the MetaNetX metabolic reaction dataset. The comparison model for bioretrosynthesis prediction is MLP with only three layers: input layer, hidden layer and output layer. The input layer is the ECFP, while the output is the label of the template. The result is shown in Table 8.

**Table 8:** Comparisons of average accuracies (%) against MLP for bioretrosynthesis on the MetaNetX dataset

Model	Top-k accuracy %			
	1	3	5	10
MLP	43.7	63.4	71.8	80.3
CNN-TMN	**45.2**	**67.0**	**73.6**	**82.2**

The best result are indicated in bold

The results in the table show that there is a 15.9% decrease in performance on the bioretrosynthesis prediction compared to the chemical retrosynthesis prediction. As shown in Fig. 3, the product lengths in the MetaNetX dataset are generally shorter than USPTO-50k in terms of the distribution of product lengths, and also in terms of the number of labels, the MetaNetX dataset is more numerous, which leads to fewer product features and more difficult predictions in MetaNetX. However, CNN-TMN still outperforms MLP by 1.6%. This also proves the effectiveness of CNN-TMN. Meanwhile, the above results provide a baseline for bioretrosynthesis prediction.

## Conclusion and discussion

### Conclusion

In this paper, we propose a new end-to-end model, termed CNN-TMN, for retrosynthesis prediction. CNN-TMN utilizes a newly designed CNN-based feature extraction network to extract multi-scale features of molecular descriptors. Specifically, in our network, we only focus on the low-level features of the sequence, which is different from the traditional CNN, which extracts the depth features. By using filters of different sizes, we can extract scalable features. In CNN-TMN, we use a two-branch feature extraction layer to extract multi-scale features of multiple molecular descriptors of a molecule and subsequently concatenate them together to obtain the final fused features for retrosynthesis prediction. The experimental results indicate that the proposed feature extraction network can effectively extract the multi-scale features of molecular descriptors, and the prediction accuracy of CNN-TMN on USPTO-50k is significantly higher than that of other existing models, demonstrating the effectiveness of CNN-TMN. In addition, we applied CNN-TMN to bioretrosynthesis prediction and provided a baseline on the MetaNetX dataset.

### Discussion

CNN-TMN proposed in this paper fully considers multiple molecular descriptors, which can make fuller use of the molecular features. The existing models consider only the molecular sequential descriptor, such as SMILES [6, 10, 12], molecular graph [7, 13] or molecular fingerprint [5]. The experiment in “Ablation study” section demonstrates that the utilization of several molecular descriptors can significantly improve the accuracy of retrosynthesis prediction. Based on the characteristics of molecular descriptors, we designed a suitable feature extraction network. In the feature extraction network, we used multiple filters of different sizes instead of fixed size, and this approach can better capture the long dependencies of sequences. Tables 5 and 6 verify the superiority of our feature extraction network. Our CNN-TMN is a template-based model, i.e., for a particular product, a template is predicted which can be applied to the product. If the predicted template does not match the ground-truth, the prediction will be considered a failure, but it is possible that the predicted templates can be applied to the product to get potential reactants. Compared to the wrong reactants predicted by a template-free model, the wrong reactants predicted by a template-based model will be more explanatory in terms of chemical principles.

However, since we consider the retrosynthesis prediction problem as a multi-classification problem and with an excessive number of classes, it is more sensitive to the impacts of the training set than other models. As shown in the experimental results in Table 5, the similar model will have a much lower prediction accuracy if the labels in the training set are not balanced, with an 11.4% decrease in the average Top-k accuracy. To make the prediction better, a splitting strategy that makes the categories more balanced, such as the method in “The Datasets” section, needs to be considered.

## Data Availability

The datasets and codes used during the current study are available at Code and Datasets.

## Footnotes

1 EBMs is a preprint version and not officially published.
